# Health Outcomes Associated With Internalizing Problems in Early Childhood and Adolescence

**DOI:** 10.3389/fpsyg.2019.00060

**Published:** 2019-01-25

**Authors:** Matthew R. Jamnik, Lisabeth F. DiLalla

**Affiliations:** ^1^Department of Psychology, Southern Illinois University Carbondale, Carbondale, IL, United States; ^2^Family and Community Medicine, Southern Illinois University School of Medicine, University School of Medicine, Carbondale, IL, United States

**Keywords:** preschoolers, adolescence, internalizing problems, negative emotionality, health outcomes

## Abstract

Physical and mental health problems are becoming more common among the general population. Studies examining mental and physical health often indicate that mental illness early in life is associated with more detrimental health outcomes later. However, additional work is needed to better identity which psychological problems may contribute to poorer health outcomes. Given recent increases in childhood anxiety and depression specifically, it is beneficial to further investigate the relationship between internalizing problems, both early and later in life, and related health problems. Furthermore, little work has focused on studying internalizing problems in children as young as preschool-aged. Therefore, the current project used a longitudinal design to assess the effects of preschool and adolescent internalizing problems on health-related problems in adolescence. We analyzed data from 70 youth (47% male) who had been tested in our lab when they were 5 years old and then were administered questionnaires over a telephone interview when they were adolescents, between the ages of 12 and 20 years old. We used multi-informant measures, including parent-report at age 5 and youth-report at follow-up, 7 to 15 years later. Parents reported on children’s internalizing behavior problems and negative emotionality (NE). Youth reported on their own internalizing behavior problems as well as health problems, physical activity, and overeating behaviors. Path modeling was used to examine predictions of internalizing and health behaviors. At age 5, parent-reported NE and internalizing problems were related, in addition to 5-year-old internalizing predicting health problems and overeating at follow-up. At follow-up, youth-reported internalizing was positively related to health problems and negatively related to physical activity, suggesting some similarities and differences between parent and youth responses. Additionally, girls reported significantly higher rates of internalizing and health problems at follow-up. These results indicate a significant relationship between preschool-aged and adolescent internalizing problems and related health outcomes experienced in adolescence.

## Introduction

According to the U.S. Department of Health and Human Services (HHS), physical health problems among the general population are dramatically increasing, as obesity is rising and many fail to meet their daily physical activity and nutrition requirements (U.S. Department of Health and Human Services [HHS], 2017). This is particularly alarming regarding children and adolescents, as these populations display increased likelihoods of poorer health later in life (Ramsawh et al., 2011). Young people today have an elevated risk for high body mass index (BMI; Bradley et al., 2008; Brook et al., 2009) and decreased activity (Zach et al., 2013), which may then cause other physical impairments. In fact, one study found that only about 10% of adolescents met physical activity guidelines set by the World Health Organization [WHO] (2004) (Zach et al., 2013). Their guidelines stated that individuals should do approximately 30 min moderate-intensity daily physical activity, which is even less than current recommendation for 60 min of moderate- to vigorous-intensity daily exercise made by the HHS (U.S. Department of Health and Human Services [HHS], 2017).

These health-related risks are particularly worrisome because they contribute significantly to school absenteeism (Hopkins et al., 2013) and are steadily increasing among children (Centers for Disease Control and Prevention [CDC], 2015a). Identifying possible stressors that may contribute to these health risks is important. Therefore, the current project assessed the relationship between adolescent health outcomes (i.e., physical activity and dietary habits, days spent ill and overall health symptoms, and BMI) and internalizing problems in preschoolers and adolescents (aged 12–20 years).

In addition to physical health problems, nearly half of Americans are affected by mental health disorders (Anxiety and Depression Association of America [ADAA], 2015). According to the World Health Organization [WHO] (2013), these problems are a major source of disability and are commonly experienced as anxiety and/or depression. Considering that childhood mental illness is a substantial predictor of adulthood psychopathology (Hofstra et al., 2002; Kessler et al., 2007; Moffitt et al., 2007), mental health early in life deserves critical attention (Caserta et al., 2011). Anxiety and depression among children have increased in recent years (National Alliance on Mental Illness, 2014) and may be associated with increased general medical disorders (Kessler and Greenberg, 2002).

Nearly 9% of preschoolers experience symptoms of anxiety and 2% experience depression (Wichstrøm et al., 2012). By adolescence, these rates increase to approximately 25 and 10%, respectively (Kessler et al., 2012). Other studies have found similar patterns, suggesting that anxiety disorders occur in high prevalence in children as young as 3 years old (Egger and Angold, 2006) and frequently co-occur with depressive symptoms throughout early childhood and adolescence (Sterba S. et al., 2007; Sterba S.K. et al., 2007). Nevertheless, research has often targeted middle childhood and adolescence (Galambos et al., 2003) and has failed to focus on younger children. Hence, we focused on preschoolers’ internalizing problems and related health outcomes in adolescence.

### Mental and Physical Health

Mental health is conceptualized as the individual’s psychological and emotional state, whereas physical health refers to their biological and somatic state (e.g., illness and disease). For this project, factors that are influenced by an individual’s mental and physical health are conceptualized as overall health outcomes. In this framework, ‘health’ may also be broadly thought of as one’s mental and physical well-being.

The relationship between mental health and physical health, or the mind and body, and the influence each have on one another are included within the theoretical foundation of the biopsychosocial model (Engel, 1980; Borrell-Carrió et al., 2004). This model proposes that illness (mental or physical) arises due to interactions between biological, psychological, and social factors. Therefore, examining the interplay between mental health and outcomes related to physical health is well-supported by this holistic philosophical approach.

Additional research is needed to elucidate the numerous health factors that may be affected by early psychological difficulties, including anxiety and/or depression. Mood problems in childhood are especially concerning because they have health implications that are not fully understood (Caserta et al., 2011). A significant comorbidity has been demonstrated between the experience of anxiety and/or depression and various related physical illnesses (Thomas et al., 2003; Sareen et al., 2006; Stein et al., 2006). In fact, higher levels of somatic-type symptoms (e.g., shortness of breath, nausea) are reported in those with chronic anxiety, which may be due to a greater awareness of physical feelings related to the experience of anxiety (Fees et al., 1999). Research examining the association between childhood mental illness (e.g., anxiety and depression) and later undesirable health outcomes has suggested that these factors are intertwined (e.g., Pine et al., 1998; Ramsawh et al., 2011), which is supported within the biopsychosocial model. Additionally, Sareen et al. (2006) suggest that internalizing and physical health may be connected through the autonomic nervous system (e.g., stress-induced ‘fight-or-flight’), which affects immune functioning and subsequent physical health.

Models of chronic stress include an interplay between mental health, stress, and biology (i.e., physical health). For example, the chronic stress paradigm indicates that mental health stressors may increase vulnerability to illnesses by weakening the immune system, thereby increasing susceptibility to developing health problems (Cohen et al., 1998), although the reverse may also occur (Menesini et al., 2009). Also, the allostatic stress model (McEwen, 2000; Juster et al., 2010) refers to the allostatic load (i.e., the “wear and tear on the body”) that accumulates in response to chronic stressors the individual experiences and reflects the physiological consequences of repeated exposure (McEwen, 2000).

Together, these models support the hypothesis that mental health (e.g., stress, anxiety) can significantly impact physical health. However, additional work is needed to better identify which psychological problems may contribute to poorer health outcomes. Related to the current investigation, previous results have indicated that increased stress is related to heightened internalizing in adolescence (Grant et al., 2004). Thus, experiencing internalizing problems may be stressful for the individual and, in turn, these problems then contribute to poorer physical health outcomes.

### Internalizing Problems and Health

Examining the impact of internalizing problems (including anxiety or depression) across the lifespan has important public health implications (Caserta et al., 2011). Given that internalizing problems in first grade are predictive of similar problems later (Essex et al., 2009) and adolescent internalizing problems predict poorer health and depression in middle adulthood (Herrenkohl et al., 2010), studying these problems in young children is beneficial to best inform early prevention and intervention efforts.

Research investigating biological factors associated with internalizing problems provides support for the proposed association between mental and physical health. For instance, significant differences in biological immune functioning have been demonstrated as a function of children’s depression (Bartlett et al., 1995; Caserta et al., 2011). Similarly, internalizing problems at age 8 significantly predicted elevated inflammatory markers at age 10; however, age 10 inflammatory markers were not significantly predictive of age 12 internalizing (Slopen et al., 2013). Relatedly, adolescent children with chronic illness self-reported greater internalizing problems than those with no chronic illness (Woods et al., 2012). Internalizing and mood problems have been related to heightened risk for physical health symptoms (e.g., infectious diseases, respiratory illnesses, risk behavior-related health problems, and weight problems) in children ages 8 to 20 (Aarons et al., 2008; Biebl et al., 2011; Nelson et al., 2013) and may lead youth to engage in certain behaviors that negatively influence their health (Biebl et al., 2011).

Other work investigating negative health outcomes similar to those examined here (e.g., problematic eating behaviors, absenteeism, and BMI) also emphasize the harmful effects of internalizing on health. For example, internalizing symptoms were significantly correlated with attitudes and behaviors related to disordered eating (Chardon et al., 2016). Additionally, a review paper concluded that two prevalent contributors to children’s problematic absenteeism commonly include depression and anxiety (Kearney, 2008). Other work supports the bidirectional relationship between internalizing problems and higher BMI (Bradley et al., 2008; Brook et al., 2009). Thus, there is general support for the interactive relationship between mental and physical health, or the biopsychosocial approach to overall health outcomes.

The role of temperament on internalizing behaviors is also important to examine, as temperament is broadly defined as biologically-based individual differences in emotionality, activity, attention, and self-regulation (Rothbart, 1989). Hence, temperament may have an influence on differences in the relationship between mental and physical health (i.e., internalizing and health outcomes, as proposed here). Several studies have indicated a relationship between child temperament and subsequent health variables (e.g., physical activity and dietary habits; Luby et al., 2002; Tucker et al., 2011). One relevant temperamental construct is negative emotionality (NE), which includes negative emotions such as anger, irritability, and frustration (Deater-Deckard and Wang, 2012). This emerges early in infancy (Rothbart, 1989) and may be related to preschool-aged externalizing and internalizing problem behaviors (Gartstein et al., 2012). Therefore, incorporation of a measure of NE will enhance our understanding of the relationship between internalizing and health outcomes.

### The Current Study

To date, little research has investigated the impact of early internalizing problems on concurrent and subsequent physical health, especially in preschool-aged children, despite previous research indicating the negative impact of internalizing problems early in life (Pine et al., 1998; Luby et al., 2003; Biebl et al., 2011; Ramsawh et al., 2011). Examining these problems in preschoolers is especially important, given the elevated risks associated with increased trajectories for internalizing throughout life (Essex et al., 2009). Additionally, investigating possible sex differences is necessary because previous research has suggested that boys and girls experience internalizing differently (Leadbeater et al., 1999).

Thus, one goal of the current study was to use a longitudinal design to evaluate the effects of preschool and adolescent internalizing problems on health-related problems in adolescence. Additionally, the role of NE on internalizing problems was also examined. We hypothesized that parent-reported internalizing problems during preschool would significantly predict youth-reported internalizing problems experienced in adolescence. We further hypothesized that parent-reported NE at age 5 would positively relate to internalizing at both time-points. Finally, we hypothesized that internalizing problems would significantly predict several negative health outcomes (e.g., poor exercise habits, problematic eating behaviors, increased somatic symptoms, number of days spent ill, and higher BMI). Sex differences were also examined, as internalizing problems tend to be greater for girls than boys (Achenbach and Edelbrock, 1979; Shin et al., 2012). We investigated these predictions using a multi-informant design, assessing parent-reported internalizing problems at preschool and youth-reported internalizing problems during adolescence. This approach allows for the compilation of a more accurate overall picture of the child from the different perspectives provided by each informant (Offord et al., 1996).

## Materials and Methods

### Participants

Participants included children who were recruited as part of the longitudinal Southern Illinois Twins/Triplets and Siblings study (DiLalla, 2002; DiLalla et al., 2013), all of whom were from rural areas and lived within a 2-h drive of the study location. For the current study, data initially were collected for 326 children tested at age 5 during the years 1994 to 2001. In 2009, 283 of these children had addresses we could locate, and these families were contacted and asked to participate in a follow-up study assessing physical and behavioral problems. Of those 283 children, 70 children (24.7%; 33 boys and 37 girls, all Caucasian; 10 twin and 10 sibling pairs, 30 singletons) now aged 12–20 years (mean age = 16.5, *SD* = 2.33) were able to be located and agreed to participate in a follow-up telephone interview. Prior to data collection, each study was approved by the Institutional Review Board (IRB).

The final sample of 70 children was compared to the original non-participating 256 participants who had data on measures relevant to this study. The number of boys and girls did not differ between families who participated in the follow-up and those who did not [χ^2^(1) = 1.15, *p* = 0.284]. Follow-up families reported significantly higher education scores for fathers, *F*(1,295) = 15.22, *p* < 0.001, and mothers, *F*(1,295) = 11.31, *p* = 0.001, and higher paternal occupation scores, *F*(1,295) = 5.43, *p* = 0.021, compared to families who did not participate. However, there were no significant differences in occupation ratings for mothers, *F*(1,295) = 0.03, *p* = 0.867. A second MANOVA indicated no significant group differences for internalizing problem behaviors, *F*(1,312) = 0.08, *p* = 0.775, or NE, *F*(1,312) = 0.53, *p* = 0.468.

### Time 1 (Age 5) Procedure and Measures

#### Procedure

Children were tested within 2 months of their fifth birthdays. Parents were contacted and invited to a laboratory playroom on campus to participate in the study. Prior to testing, parents were mailed questionnaires that they were asked to bring back on the day of testing. Parents provided signed consent for the children and for themselves prior to the advent of the study. Children participated in a free play study in the lab, but only parent questionnaires were included in the present study. All children were given toys to thank them for participating.

#### Demographic Questionnaire

A parent-report demographic questionnaire was administered at age 5 to obtain information regarding age, relation to the child, race, education, and occupation, as well as family structure and income. Parent education was rated on a five-point scale (1 = *no high school degree*; 2 = *high school degree*; 3 = *technical training and certificate*; 4 = *college degree*; 5 = *advanced degree beyond college degree*). The Hollingshead Index was used to rate parent occupations (1 = *high level professional*, to 7 = *unskilled labor*; Bonjean et al., 1967). Family income was rated on a 12-point scale (1 = *less than $5000*, to 12 = *greater than $55,000*). In the current sample, median education was 4 (college degree) for mothers (range: 2–5) and fathers (range: 1–5). Maternal and paternal occupations had medians of 4 (small business owners/skilled manual workers; range: 1–7) and 3 (technicians/semiprofessionals/small business owners; range: 1–7), respectively. The median yearly family income score was 10 ($45,000–$50,000; range: 2–12).

#### Internalizing Problems

The Child Behavior Checklist (CBCL) is a 113-item parent report form that assesses the social, emotional, and behavioral functioning of preschoolers (Achenbach and Rescorla, 2001). Parents rated their child on each item over the past 6 months using a three-point scale (0 = *not at all like my child* to 2 = *very much like my child*). The current study used the second order factor of internalizing problem behaviors, which is composed of subscales Withdrawn/Depressed, Anxious/Depressed, and Somatic Complaints. Internalizing has demonstrated high test–retest reliability (*r* = 0.91) and excellent internal consistency (Achenbach and Rescorla, 2001). Within the current sample, the internalizing scale has good internal consistency (Cronbach’s α = 0.82).

#### Negative Emotionality

Parents completed the Behavioral Style Questionnaire (BSQ; McDevitt and Carey, 1978), a 100-item rating scale designed for children ages 3 to 7 years, which provides scores on nine general temperament categories. These nine categories correspond with the nine dimensions of temperament identified in the New York Longitudinal Study (NYLS; Thomas and Chess, 1977): Activity Level, Rhythmicity, Adaptability, Approach/Withdrawal, Threshold, Intensity, Mood, Distractibility, and Persistence. Each item is answered on a six-point scale, ranging from 1 (*almost never*) to 6 (*almost always*). We created a score for NE by averaging scores on the Adaptability, Intensity, and Mood scales. NE includes items such as “The child is slow to adjust to changes in household rules,” “The child responds intensely to disapproval,” and “The child becomes angry with his/her playmates.” The scales comprising the NE score in the current sample show adequate internal reliability (Cronbach’s α = 0.74). Validity of NE has been demonstrated at age 5 (Bersted and DiLalla, 2016); NE was significantly correlated with parent-reported externalizing and internalizing problem behaviors.

### Follow-Up Procedure (Ages 12–20) and Measures

#### Procedure

At follow-up, packets describing a telephone-administered questionnaire were sent to 70 families who agreed to participate and returned signed consent forms. Included was a letter describing the purpose of the study and answer cards for the children to use during the telephone interview (each card was a different color to make it easy for children to know which card to use for the different questionnaires during the telephone interview). The interviewer received verbal consent via the telephone before administering the 45-min interview, telling the youth which color answer card to use for each questionnaire. We took several precautions against youth bias based on having someone around who could hear them during the interview: we were careful to tell parents not to be present during the interview; we asked adolescents to go to a private place; and we only had adolescents respond with answer numbers so that even if anyone were listening they would not know what was being asked of the youth. After completion, participants were verbally debriefed and then mailed a letter and gift card for $30 to thank them.

#### Internalizing Problems

The Strengths and Difficulties Questionnaire (SDQ) was used to measure participants’ internalizing problems. The SDQ is a widely used, brief, self-report screening questionnaire that measures social, emotional, and behavioral functioning across five subscales: conduct problems, emotional symptoms, hyperactivity, peer problems, and prosocial behaviors (Goodman, 1997, 2001). Youth responded to 25 items using a three-point scale (0 = *not true* to 2 = *certainly true).* Support has been demonstrated for combining these subscales in non-clinical samples to create two second-order factors, internalizing (emotional symptoms and peer problems) and externalizing (conduct problems and hyperactivity) problems (Goodman et al., 2010). Thus, the current project averaged the emotional and peer subscales to create an overall construct of internalizing problems. Reliability estimates for the current study were adequate and similar to the original sample (Goodman, 2001), 0.61 for emotional problems, 0.42 for peer problems. Although the reliability was low for peer problems, we maintained these items because the overall internalizing scale showed adequate reliability (Cronbach’s α = 0.62) and because the second-order internalizing scale has been recommended for non-clinical samples.

#### Physical Health Problems

Physical health was assessed using two different questionnaires. The Physical Health Questionnaire (PHQ) is a 14-item health scale that provides an overall rating of physical health problems (Schat et al., 2005). Youth rate each item on a seven-point scale, from 1 = *Not at all* to 7 = *All the time*, assessing how often they experienced these symptoms over the last 6 months. Items assess all types of physical health, such as “How often have you woken up during the night?” and “How often have you experienced headaches?” The PHQ has adequate internal consistency and construct validity (Schat et al., 2005). For the Overall Physical Health Problems scale, which was used for our analyses, internal consistency reliability for our sample was adequate, Cronbach’s α = 0.77.

The Health Problems Checklist (HPC) is a health measure that was created to measure the severity and chronicity of common health problems (Wonderlich, 2007) and was modeled after a checklist examining the mental and physical health of youth (Aarons et al., 2008). Twenty-seven items asked about specific illnesses, but these items were not used in the present analyses because they were comparable to the PHQ scale, which was used instead. Then youth responded to three items rated on a five-point scale: “How often do you try to avoid P.E. or extracurricular athletic activities?” “Frequency of eating large amounts of food without regard to quantity eaten” and “Frequency of eating large amounts of food when feeling upset or out of control.” Youth also were asked to report the total number of days spent ill in the last year. Finally, youth were asked how many days per week they engaged in intense physical activity (“where your heart beats faster and you’re breathing harder than normal for 30 min or more”) during the past week. These five items were used in our analyses.

#### Body-Mass Index (BMI)

Participants self-reported their height and weight. Overall BMI was calculated by dividing weight (kg) by height (in meters) squared. To account for developmental differences that occur due to age and sex, it is important to use normed BMI scores. These categories are based on separate guideline recommendations for boys and girls provided by the Centers for Disease Control and Prevention (Centers for Disease Control and Prevention [CDC], 2015b) for assessing BMI in children.

### Data Analytic Plan

**FIGURE 1:**
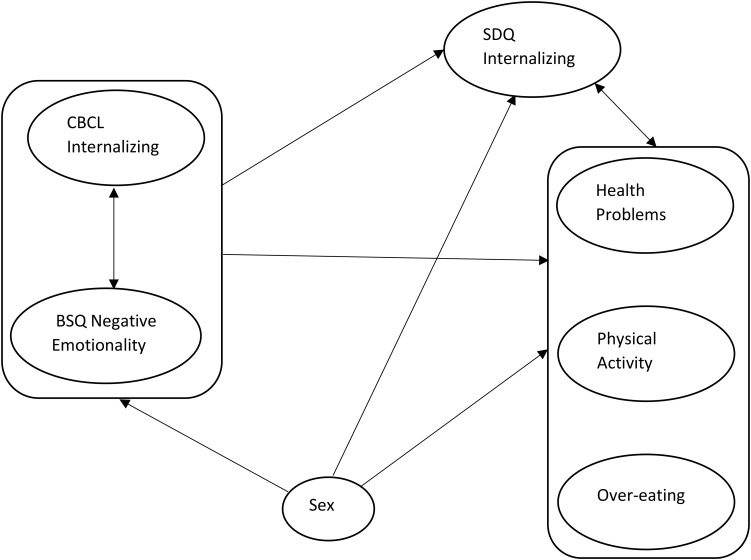
Full conceptual path analysis model.

Prior to examining our full model, we computed a factor analysis on the health variables to combine them into cohesive factors. We used these factors in the remaining analyses. Then, to examine the relationships between age 5 problems (negative emotionality and internalizing) and internalizing and health problems at follow-up, we used LISREL (Joreskog and Sorbom, 1996) to conduct a path model analysis that allowed us to consider all measures simultaneously. We ran a correlation matrix between all study variables for input to the path analysis, and we also ran the correlation matrix randomly omitting one sibling from each family (*n* = 50). This matrix was nearly identical to the full-sample matrix, so we were comfortable using the full sample in analyses. For the path analysis, we allowed the age 5 variables of internalizing and NE to be correlated, and then we examined predictions from age 5 to internalizing and health outcome measures and from sex to all measures, and correlations between follow-up internalizing and the three health factors (see Figure 1). We then tested nested models by dropping (1) paths from temperament to follow-up measures; (2) paths from sex to 5-year-old measures; (3) paths from sex to follow-up measures; (4) other paths to internalizing and health outcomes to determine the most parsimonious model.

## Results

First, variables were examined for skewness. The only measure that displayed extreme skewness was average number of days sick. We cube rooted that measure to reduce skewness and then used that transformed variable in analyses. Table 1 displays demographic characteristics for all variables.

**Table 1:** Demographic characteristics for all study variables.

Variable	Mean	*SD*	Actual range	Possible range
Negative emotionality	3.32	0.55	2.31 to 4.64	1 to 5
CBCL internalizing age 5	4.89	5.05	0 to 23	0 to 64
SDQ internalizing follow-up	2.10	1.37	1 to 2.10	1 to 3
PHQ overall physical health problems	2.61	0.54	1.71 to 3.86	1 to 7
Eats without thinking	2.46	1.07	1 to 5	1 to 5
Emotional eating	1.60	0.79	1 to 4	1 to 5
Avoids physical activity	1.37	0.77	1 to 4	1 to 5
Physical activity in past week	3.70	2.13	0 to 7	0 to 7
Average days sick, past year	6.94	14.03	0.5 to 108.5	0 to 356
Transformed average days sick	1.64	0.64	0.79 to 4.77	0 to 7.09
BMI	22.40	4.46	16.2 to 41.3	
Health problems factor 1	0.00	0.75	−1.14 to 2.78	
Physical activity factor 2	0.00	0.82	−2.70 to 1.10	
Overeating factor 3	0.00	0.73	−1.24 to 2.37	

### Factor Analysis

We performed a principal components factor analysis with varimax rotation to determine factors for the health outcome variables. We found that a three-factor rotated solution accounted for 64% of the total variance (see Table 2). The three factors represent Health Problems, Physical Activity, and Overeating. Items loading greater than 0.50 were z-scored (to account for different scaling) and averaged to create three factor scores for further analyses.

**Table 2:** Factor loadings greater than 0.50 for all health variables.

Item	Factor 1 (health problems)	Factor 2 (physical activity)	Factor 3 (overeating)
PHQ overall health problems	0.82		
Transformed – average days sick	0.74		
Emotional eating	0.60		
Avoids physical activity		0.80	
Physical activity in past week		0.75	
Eats without thinking			0.74
BMI			0.64

### Path Analyses

A correlation matrix showing the inter-correlations between all variables included in the path analysis is presented in Table 3. Using LISREL, we first ran our full model, which allowed for a correlation between the 5-year-old variables and included paths from both 5-year-old variables to all follow-up variables. It also included correlations between follow-up internalizing and the three health factors. Finally, a path from sex to the other six variables was freed. This full model provided a relatively good fit to the data (see Table 4), χ^2^(3) = 2.46, *p* = 0.482, BIC = 526.185, CFI = 1.00, RMSEA = 0.0.

**Table 3:** Correlation matrix of all study variables.

	CBCL internalizing	BSQ negative emotionality	SDQ internalizing	Health problems	Physical activity	Overeating	Sex
CBCL internalizing	1.0						
BSQ negative emotionality	0.36^∗∗^	1.0					
SDQ internalizing	0.03	0.06	1.0				
Health problems	0.37^∗∗^	0.10	0.28^∗^	1.0			
Physical activity	0.11	−0.11	−0.45^∗∗∗^	0.05	1.0		
Overeating	0.29^∗^	0.08	0.03	0.15	0.01	1.0	
Sex (0 = girl; 1 = boy)	−0.07	−0.03	−0.28^∗^	−0.37^∗∗^	0.19	0.19	1.0

*^∗^p < 0.05; ^∗∗^p < 0.01; ^∗∗∗^p < 0.001.*

**Table 4:** Model fit statistics.

	X^2^(df)	*p*	RMSEA [CI]	CFI	Compared to model	ΔC^2^(df)	*p*	BIC
(1) Full model	2.46 (3)	0.482	0.00 [0.000; 0.187]	1.000				526.185
(2) No paths from negative emotionality	4.33 (7)	0.741	0.00 [0.700; 0.778]	1.000	1	1.87 (4)	ns	511.062
(3) No paths from sex to age 5	4.68 (9)	0.608	0.00 [0.000; 0.072]	1.000	2	0.35 (3)	ns	502.911
(4) No paths from sex to follow-up	22.08 (13)	0.054	0.10 [0.000; 0.169]	0.824	3	13.87 (3)	<0.005	503.312
(5) Model 3 + drop all ns paths	12.40 (14)	0.574	0.00 [0.000; 0.104]	1.000	3	7.72 (5)	ns	489.389

*RMSEA, root mean square error of approximation; CFI, Comparative Fit Index; BIC, Bayesian Information Criterion.*

**FIGURE 2:**
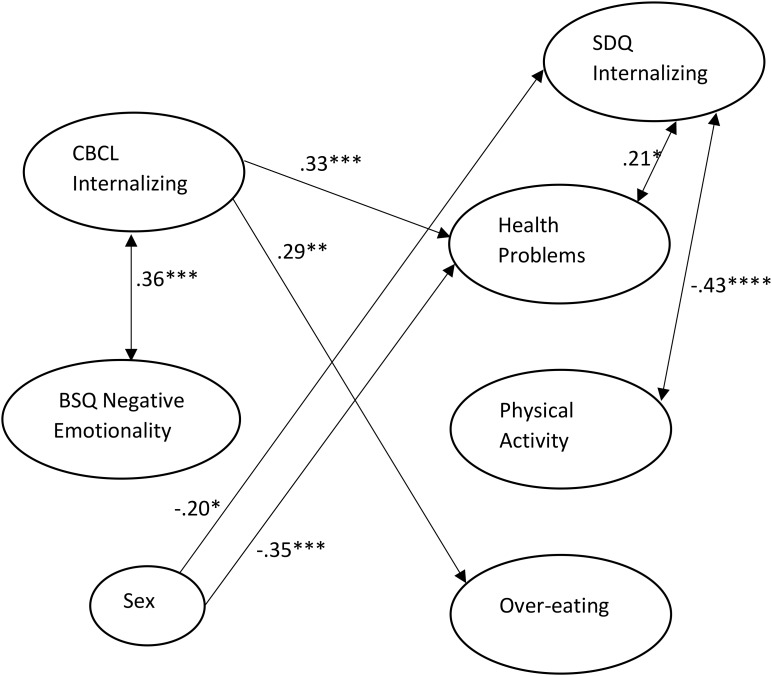
Final path model with only significant paths included in Figure. Path estimates are standardized. All residual variances were fixed at 1. ^∗^*p* < 0.025; ^∗∗^*p* < 0.01; ^∗∗∗^*p* < 0.005; ^∗∗∗∗^*p* < 0.001.

For Model 2, we fixed to zero all paths from NE to follow-up variables. This did not result in a significant decrement of model fit and the BIC index was smaller, χ^2^(7) = 4.33, *p* = 0.741, BIC = 511.062, CFI = 1.00, RMSEA = 0.0, so these paths were omitted from the model. For Model 3, we dropped all paths from sex to the 5-year-old variables. This did not result in a significantly worse fit, χ^2^(10) = 8.21, *p* = 0.608, BIC = 502.193, CFI = 1.00, RMSEA = 0.0. For Model 4, we dropped the remaining paths from sex to all follow-up variables, and this did result in a significantly worse fit, χ^2^(13) = 22.08, *p* = 0.054, BIC = 503.312, CFI = 0.824, RMSEA = 0.100, so those paths were retained in the model. Finally, in order to rigorously assess the significance of each of the remaining seven paths as well as the paths from sex to the follow-up variables, they were dropped one at a time from the model. The final model is shown in Figure 2 and only includes paths that were significant.

This best fitting model demonstrates a significant correlation between internalizing and NE at age 5. Girls scored higher on self-reported internalizing and health problems at follow-up. Internalizing at age 5 significantly predicted self-reported health problems and overeating, whereas adolescent internalizing was positively correlated with concurrent health problems and negatively correlated with physical activity.

## Discussion

Considering the rising prevalence of physical health problems among the general population (U.S. Department of Health and Human Services [HHS], 2017), as well as an increase in health-related problems among children (Centers for Disease Control and Prevention [CDC], 2015a), it is important to further elucidate mechanisms that may be related to these problematic health outcomes. This point is further emphasized by other work displaying an increase in mental health difficulties, such as anxiety and depression, among children in recent years (National Alliance on Mental Illness, 2014). Despite this, many studies have failed to examine these relationships longitudinally, especially beginning in early childhood. Therefore, the present study adds to the literature by investigating the relationship between internalizing problems, both in early childhood and at follow-up, and adolescent health outcomes. We used the biopsychosocial model as a framework and found that mental and physical health were related in our sample.

Using a longitudinal design, our study assessed the relationship between preschool-aged internalizing problems and NE predicting follow-up health problems in adolescence. Data from multiple informants were collected, using both parent-report questionnaires (at age 5) and youth-reported measures (at adolescence), in order to accurately capture the problems under investigation. Overall, our path model indicated an overarching relationship between internalizing problems and health outcomes. That is, we found that increased internalizing problems were related to heightened health problems across both informants and time points.

Specifically, we found a significant positive relationship between age 5 NE and internalizing problems, indicating that increased internalizing problems may be related to simultaneous aspects of temperament such as anger, irritability, and frustration (i.e., negative emotionality). This supports previous research that found similar results (Gartstein et al., 2012), although in our sample 5-year-old temperament was not related to adolescent internalizing problems. Additionally, we found that parent-reported internalizing at age 5 significantly predicted increased overeating and health problems as reported by adolescents. We also found that youth-reported internalizing problems were related to negative health outcomes. That is, increased internalizing at follow-up was positively related to health problems and negatively related to physical activity behaviors. Thus, we found some shared overlap between both parent-reported and youth-reported internalizing problems and adolescent health problems, as well as distinctions between each of these internalizing measures. That is, age 5 internalizing problems predicted increased overeating, whereas follow-up internalizing problems related negatively to physical activity. Finally, our model also indicated a sex effect, with girls scoring higher on both internalizing and health problems at follow-up.

Surprisingly, we did not find an association between parent-reported and youth-reported internalizing problems. This lack of association is partly a result of using different measures at each time point (CBCL versus SDQ) as well as the fact that the time period between measurements was large, ranging from 7 to 15 years apart. Additionally, differences in informant perceptions of internalizing have been noted (Sourander et al., 1999), although they are not usually this extreme (Vierhaus et al., 2018). Nonetheless, we believe that our multi-method, multi-informant means of collecting data maximized our ability to assess internalizing optimally at each age.

Overall, these results provide additional evidence for the body of literature supporting the biopsychosocial model (Engel, 1980) for investigating health outcomes (Borrell-Carrió et al., 2004). Our study also found evidence for a relationship between mental and physical health, as have other studies (e.g., Pine et al., 1998; Herrenkohl et al., 2010). Like previous research, we also demonstrated a relationship between internalizing problems and eating behavior (Chardon et al., 2016), days spent sick (Kearney, 2008; Hopkins et al., 2013), and general somatic symptoms (e.g., illness; Woods et al., 2012). Like Gartstein et al. (2012), we did find a relationship between temperament and internalizing problems at age 5, but temperament did not predict internalizing in adolescence. In general, our findings did suggest that increased internalizing is related to negative health outcomes, which supports other studies with similar results (e.g., Luby et al., 2003; Biebl et al., 2011; Ramsawh et al., 2011).

### Strengths and Limitations

The results of the present study differ from previous research and add to the literature in several important ways. First, a multi-informant design was used to capture internalizing problems most accurately across development, which is in line with work from other researchers (Offord et al., 1996). At age 5, preschoolers do not yet have the cognitive capabilities to recognize their own internalizing behaviors and, therefore, parent report is necessary. However, by adolescence, youth may interpret their own internalizing problems best and, thus, youth-report is likely most informative at that age. The similarities and differences seen between internalizing and health outcomes, dependent on the informant and age of measurement, highlight these considerations. Next, we focused on a variety of health outcomes (i.e., health problems, physical activity, and overeating) that may be unique to the current study. Past research has often examined health measures that were more biologically based (e.g., inflammatory markers and chronic illnesses; Caserta et al., 2011; Slopen et al., 2013; Woods et al., 2012), rather than the more subjective measures (e.g., days feeling ill, physical activity avoidance, emotional eating) used in the current study. Finally, the relationship between preschoolers’ internalizing problems and health outcomes many years later was specifically emphasized. Examining these problems early in life is especially important, given that little work has focused on preschool-aged children, despite many studies indicating elevated risks associated with early-life internalizing (Pine et al., 1998; Luby et al., 2003; Essex et al., 2009; Biebl et al., 2011; Ramsawh et al., 2011).

Strengths notwithstanding, a few limitations in the current study must be put forth. For one, our study relied on youth reporting their own health problems at follow-up, in addition to internalizing problems, which may have inflated the concurrent relationships described between each. It could be that youth overestimated their experiences of internalizing or health problems. Despite this possibility, our findings indicating that age 5 internalizing also predicted follow-up health problems helps mitigate this concern. Additionally, using a self-report measure of various health-related behaviors (i.e., health problems, physical activity, and overeating) seemed most beneficial for this project because we argue that the individual’s perceptions and experiences of health problems, as opposed to the accuracy of these problems, are most relevant to internalizing problems.

## Conclusion

This study examined possible influences on health outcomes in adolescence by focusing on past internalizing problems (at age 5) reported by parents and concurrent internalizing problems reported by youth at follow-up (ages 12–20). Supporting past research, our study found that elevated internalizing problems were associated with heightened negative health outcomes, regardless of age measured. Furthermore, we found unique distinctions in the relationships between internalizing and health, dependent on the informant questioned. These results highlight the importance of collecting measures from multiple informants to assess the construct under investigation most thoroughly. Finally, by examining preschoolers, we hoped to emphasize the necessity of investigating these problems and their influences on health in a younger age than typically studied. Given the rise of mental health and physical health problems early in life (Centers for Disease Control and Prevention [CDC], 2015a) and their impact on health later in life (Ramsawh et al., 2011), it is critical to begin recognizing and addressing these problems at the youngest age possible. Focusing future work on aiming to alleviate internalizing problems early in life will be one fruitful avenue for optimizing individual health outcomes later in life.

## Ethics Statement

The Southern Illinois University Human Subjects Committee (HSC) approved all data collection prior to the beginning of the study. At age 5, all children’s parents signed consent forms prior to data collection. At adolescence, all children and their parents gave verbal consent over the telephone prior to answering questionnaires over the telephone. This was approved by the HSC.

## Author Contributions

MJ and LD contributed conception and design of the study and wrote sections of the first draft of the manuscript. MJ organized the database. LD performed the statistical analysis. All authors contributed to manuscript revision, read and approved the submitted version.

## Conflict of Interest Statement

The authors declare that the research was conducted in the absence of any commercial or financial relationships that could be construed as a potential conflict of interest.

## References

[B1] Aarons G. A. Monn A. R. Leslie L. K. Garland A. F. Lugo L. Hough R. L. (2008). Association between mental and physical health problems in high-risk adolescents: a longitudinal study. *J. Adolesc. Health* 43 260–267. 10.1016/j.jadohealth.2008.01.013 18710681 PMC2768339

[B2] Achenbach T. M. Edelbrock C. S. (1979). The child behavior profile: II. Boys aged 12–16 and girls aged 6–11 and 12–16. *J. Consult. Clin. Psychol.* 47 223–233. 10.1037/0022-006X.47.2.223 469068

[B3] Achenbach T. M. Rescorla L. A. (2001). *Manual for the ASEBA School-Age Forms & Profiles.* Burlington, VT: University of Vermont.

[B4] Anxiety and Depression Association of America [ADAA] (2015). *Survey Finds that Americans Value Mental Health and Physical Health Equally: Mental Health care Viewed as Inaccessible and Unaffordable by Many.* Available at: http://actionallianceforsuicideprevention.org/sites/actionallianceforsuicideprevention.org/files/Final%20Survey%20Press%20Release_0.pdf

[B5] Bartlett J. A. Schleifer S. J. Demetrikopoulos M. K. Keller S. E. (1995). Immune differences in children with and without depression. *Biol. Psychiatry* 38 771–774. 10.1016/0006-3223(95)00364-9 8580234

[B6] Bersted K. A. DiLalla L. F. (2016). The influence of DRD4 genotype and perinatal complications on preschoolers’ negative emotionality. *J. Appl. Dev. Psychol.* 42 71–79. 10.1016/j.appdev.2015.12.001

[B7] Biebl S. J. DiLalla L. F. Davis E. K. Lynch K. A. Shinn S. O. (2011). Longitudinal associations among peer victimization and physical and mental health problems. *J. Pediatr. Psychol.* 36 868–877. 10.1093/jpepsy/jsr025 21685460

[B8] Bonjean C. M. Hill R. J. McLemore S. D. (1967). Sociological measurement: an inventory of scales and indices. *Soc. Forces* 43 532–535. 10.2307/2574460

[B9] Borrell-Carrió F. Suchman A. L. Epstein R. M. (2004). The biopsychosocial model 25 years later: principles, practice, and scientific inquiry. *Ann. Fam. Med.* 2 576–582. 10.1370/afm.245 15576544 PMC1466742

[B10] Bradley R. H. Houts R. Nader P. R. O’brien M. Belsky J. Crosnoe R. (2008). The relationship between body mass index and behavior in children. *J. Pediatr.* 153 629–634. 10.1016/j.jpeds.2008.05.026 18639889 PMC2590939

[B11] Brook J. S. Zhang C. Saar N. S. Brook D. W. (2009). Psychosocial predictors, higher body mass index, and aspects of neurocognitive dysfunction. *Percept. Mot. Skills* 108 181–195. 10.2466/pms.108.1.181-195 19425460 PMC2742374

[B12] Caserta M. T. Wyman P. A. Wang H. Moynihan J. O’Connor T. G. (2011). Associations among depression, perceived self-efficacy, and immune function and health in preadolescent children. *Dev. Psychopathol.* 23 1139–1147. 10.1017/S0954579411000526 22018086 PMC3605886

[B13] Centers for Disease Control and Prevention [CDC] (2015a). *Healthy Weight: About Child and Teen BMI.* Available at: https://www.cdc.gov/healthyweight/assessing/bmi/childrens_bmi/about_childrens_bmi.html

[B14] Centers for Disease Control and Prevention [CDC] (2015b). *School Health Profiles 2014: Characteristics of Health Programs Among Secondary Schools.* Available at: https://www.cdc.gov/healthyyouth/data/profiles/pdf/2014/2014_profiles_report.pdf

[B15] Chardon M. L. Janicke D. M. Carmody J. K. Dumont-Driscoll M. C. (2016). Youth internalizing symptoms, sleep-related problems, and disordered eating attitudes and behaviors: a moderated mediation analysis. *Eat. Behav.* 21 99–103. 10.1016/j.eatbeh.2016.01.007 26826649

[B16] Cohen S. Frank E. Doyle W. J. Skoner D. P. Rabin B. S. Gwaltney J. M. Jr. (1998). Types of stressors that increase susceptibility to the common cold in healthy adults. *Health Psychol.* 17 214–223. 10.1037/0278-6133.17.3.214 9619470

[B17] Deater-Deckard K. Wang Z. (2012). “Anger and irritability,” in *Handbook of Temperament*, eds Zentner M. Shiner R. L. (New York, NY: The Guilford Press), 124–144.

[B18] DiLalla L. F. (2002). Preschool social and cognitive behaviors: the Southern Illinois Twins. *Twin Res.* 5 468–471. 10.1375/136905202320906291 12537878

[B19] DiLalla L. F. Gheyara S. Bersted K. (2013). The Southern Illinois Twins and Siblings Study (SITSS): description and update. *Twin Res. Hum. Genet.* 16 371–375. 10.1017/thg.2012.69 23046641

[B20] Egger H. L. Angold A. (2006). Common emotional and behavioral disorders in preschool children: presentation, nosology, and epidemiology. *J. Child Psychol. Psychiatry* 47 313–337. 10.1111/j.1469-7610.2006.01618.x 16492262

[B21] Engel G. L. (1980). The clinical application of the biopsychosocial model. *Am. J. Psychiatry* 137 535–544. 10.1176/ajp.137.5.535 7369396

[B22] Essex M. J. Kraemer H. C. Slattery M. J. Burk L. R. Thomas Boyce W. Woodward H. R. (2009). Screening for childhood mental health problems: outcomes and early identification. *J. Child Psychol. Psychiatry* 50 562–570. 10.1111/j.1469-7610.2008.02015.x 19432682 PMC2682224

[B23] Fees B. S. Martin P. Poon L. W. (1999). A model of loneliness in older adults. *J. Gerontol. B Psychol. Sci. Soc. Sci.* 54 231–239. 10.1093/geronb/54B.4.P231 12382592

[B24] Galambos N. L. Barker E. T. Almeida D. M. (2003). Parents do matter: trajectories of change in externalizing and internalizing problems in early adolescence. *Child Dev.* 74 578–594. 10.1111/1467-8624.7402017 12705574

[B25] Gartstein M. A. Putnam S. P. Rothbart M. K. (2012). Etiology of preschool behavior problems: contributions of temperament attributes in early childhood. *Infant Ment. Health J.* 33 197–211. 10.1002/imhj.21312 28520102

[B26] Goodman A. Lamping D. L. Ploubidis G. B. (2010). When to use broader internalising and externalising subscales instead of the hypothesised five subscales on the strengths and difficulties questionnaire (SDQ): data from British parents, teachers and children. *J. Abnorm. Child Psychol.* 38 1179–1191. 10.1007/s10802-010-9434-x 20623175

[B27] Goodman R. (1997). The strengths and difficulties questionnaire: a research note. *J. Child Psychol. Psychiatry* 38 581–586. 10.1111/j.1469-7610.1997.tb01545.x 9255702

[B28] Goodman R. (2001). Psychometric properties of the strengths and difficulties questionnaire. *J. Am. Acad. Child Adolesc. Psychiatry* 40 1337–1345. 10.1097/00004583-200111000-00015 11699809

[B29] Grant K. E. Compas B. E. Thurm A. E. McMahon S. D. Gipson P. Y. (2004). Stressors and child and adolescent psychopathology: measurement issues and prospective effects. *J. Clin. Child Adolesc. Psychol.* 33 412–425. 10.1207/s15374424jccp3302_23 15136206

[B30] Herrenkohl T. I. Kosterman R. Mason W. A. Hawkins J. D. McCarty C. A. McCauley E. (2010). Effects of childhood conduct problems and family adversity on health, health behaviors, and service use in early adulthood: tests of developmental pathways involving adolescent risk taking and depression. *Dev. Psychopathol.* 22 655–665. 10.1017/S0954579410000349 20576185 PMC2892805

[B31] Hofstra M. B. van der Ende J. A. N. Verhulst F. C. (2002). Child and adolescent problems predict DSM-IV disorders in adulthood: a 14-year follow-up of a Dutch epidemiological sample. *J. Am. Acad. Child Adolesc. Psychiatry* 41 182–189. 10.1097/00004583-200202000-00012 11837408

[B32] Hopkins J. Lavigne J. V. Gouze K. R. LeBailly S. A. Bryant F. B. (2013). Multi-domain models of risk factors for depression and anxiety symptoms in preschoolers: evidence for common and specific factors. *J. Abnorm. Child Psychol.* 41 705–722. 10.1007/s10802-013-9723-2 23504302

[B33] Joreskog K. G. Sorbom D. (1996). *LISREL 8: Structural Equation Modeling.* Chicago, IL: Scientific Software International Corp.

[B34] Juster R. P. McEwen B. S. Lupien S. J. (2010). Allostatic load biomarkers of chronic stress and impact on health and cognition. *Neurosci. Biobehav. Rev.* 35 2–16. 10.1016/j.neubiorev.2009.10.002 19822172

[B35] Kearney C. A. (2008). School absenteeism and school refusal behavior in youth: a contemporary review. *Clin. Psychol. Rev.* 28 451–471. 10.1016/j.cpr.2007.07.012 17720288

[B36] Kessler R. C. Angermeyer M. Anthony J. C. De Graaf R. O. N. Demyttenaere K. Gasquet I. (2007). Lifetime prevalence and age-of-onset distributions of mental disorders in the World Health Organization’s World Mental Health Survey Initiative. *World Psychiatry* 6 168–176. 18188442 PMC2174588

[B37] Kessler R. C. Greenberg P. E. (2002). The economic burden of anxiety and stress disorders. *Neuropsychopharmacol. Fifth Gen. Prog.* 67 982–992.

[B38] Kessler R. C. Petukhova M. Sampson N. A. Zaslavsky A. M. Wittchen H. U. (2012). Twelve-month and lifetime prevalence and lifetime morbid risk of anxiety and mood disorders in the United States. *Int. J. Methods Psychiatr. Res.* 21 169–184. 10.1002/mpr.1359 22865617 PMC4005415

[B39] Leadbeater B. J. Kuperminc G. P. Blatt S. J. Hertzog C. (1999). A multivariate model of gender differences in adolescents’ internalizing and externalizing problems. *Dev. Psychol.* 35 1268–1282. 10.1037/0012-1649.35.5.1268 10493653

[B40] Luby J. L. Heffelfinger A. K. Mrakotsky C. Brown K. M. Hessler M. J. Wallis J. M. (2003). The clinical picture of depression in preschool children. *J. Am. Acad. Child Adolesc. Psychiatry* 42 340–348. 10.1097/00004583-200303000-00015 12595788

[B41] Luby J. L. Heffelfinger A. K. Mrakotsky C. Hessler M. J. Brown K. M. Hildebrand T. (2002). Preschool major depressive disorder: preliminary validation for developmentally modified DSM-IV criteria. *J. Am. Acad. Child Adolesc. Psychiatry* 41 928–937. 10.1097/00004583-200208000-00011 12162628

[B42] McDevitt S. C. Carey W. B. (1978). The measurement of temperament in 3–7 year old children. *J. Child Psychol. Psychiatry* 19 245–253. 10.1111/j.1469-7610.1978.tb00467.x 681467

[B43] McEwen B. S. (2000). Allostasis and allostatic load: implications for neuropsychopharmacology. *Neuropsychopharmacology* 22 108–124. 10.1016/S0893-133X(99)00129-3 10649824

[B44] Menesini E. Modena M. Tani F. (2009). Bullying and victimization in adolescence: concurrent and stable roles and psychological health symptoms. *J. Genet. Psychol.* 170 115–134. 10.3200/GNTP.170.2.115-134 19492729

[B45] Moffitt T. E. Harrington H. Caspi A. Kim-Cohen J. Goldberg D. Gregory A. M. (2007). Depression and generalized anxiety disorder: cumulative and sequential comorbidity in a birth cohort followed prospectively to age 32 years. *Arch. Gen. Psychiatry* 64 651–660. 10.1001/archpsyc.64.6.651 17548747

[B46] National Alliance on Mental Illness (2014). *Children and Teens Mental Health Facts.* Available at: https://www.nami.org/NAMI/media/NAMI-Media/Infographics/Children-MH-Facts-NAMI.pdf

[B47] Nelson T. D. Smith T. R. Duppong Hurley K. Epstein M. H. Thompson R. W. Tonniges T. F. (2013). Association between psychopathology and physical health problems among youth in residential treatment. *J. Emot. Behav. Disord.* 21 150–160. 10.1177/1063426612450187

[B48] Offord D. R. Boyle M. H. Racine Y. Szatmari P. Fleming J. E. Sanford M. (1996). Integrating assessment data from multiple informants. *J. Am. Acad. Child Adolesc. Psychiatry* 35 1078–1085. 10.1097/00004583-199608000-00019 8755805

[B49] Pine D. S. Cohen P. Gurley D. Brook J. Ma Y. (1998). The risk for early-adulthood anxiety and depressive disorders in adolescents with anxiety and depressive disorders. *Arch. Gen. Psychiatry* 55 56–64. 10.1001/archpsyc.55.1.56 9435761

[B50] Ramsawh H. J. Weisberg R. B. Dyck I. Stout R. Keller M. B. (2011). Age of onset, clinical characteristics, and 15-year course of anxiety disorders in a prospective, longitudinal, observational study. *J. Affect. Disord.* 132 260–264. 10.1016/j.jad.2011.01.006 21295858 PMC3109118

[B51] Rothbart M. K. (1989). “Temperament in childhood: a framework,” in *Temperament in Childhood*, eds Kohnstamm G. A. Bates J. E. Rothbart M. K. (New York, NY: Wiley), 59–73.

[B52] Sareen J. Jacobi F. Cox B. J. Belik S. L. Clara I. Stein M. B. (2006). Disability and poor quality of life associated with comorbid anxiety disorders and physical conditions. *Arch. Intern. Med.* 166 2109–2116. 10.1001/archinte.166.19.2109 17060541

[B53] Schat A. C. Kelloway E. K. Desmarais S. (2005). The physical health questionnaire (PHQ): construct validation of a self-report scale of somatic symptoms. *J. Occup. Health Psychol.* 10 363–381. 10.1037/1076-8998.10.4.363 16248686

[B54] Shin Y. M. Sung M. J. Lim K. Y. Park K. S. Cho S. M. (2012). The pathway of internalizing and externalizing problems from childhood to adolescence: a prospective study from age 7 to 14–16 in Korea. *Community Ment. Health J.* 48 384–391. 10.1007/s10597-011-9468-8 22089146

[B55] Slopen N. Kubzansky L. D. Koenen K. C. (2013). Internalizing and externalizing behaviors predict elevated inflammatory markers in childhood. *Psychoneuroendocrinology* 38 2854–2862. 10.1016/j.psyneuen.2013.07.012 24011503

[B56] Sourander A. Helstelä L. Helenius H. (1999). Parent-adolescent agreement on emotional and behavioral problems. *Soc. Psychiatry Psychiatr. Epidemiol.* 34 657–663. 10.1007/s001270050189 10703276

[B57] Stein M. B. Cox B. J. Afifi T. O. Belik S. L. Sareen J. (2006). Does co-morbid depressive illness magnify the impact of chronic physical illness? A population-based perspective. *Psychol. Med.* 36 587–596. 10.1017/S0033291706007239 16608557

[B58] Sterba S. Egger H. L. Angold A. (2007). Diagnostic specificity and nonspecificity in the dimensions of preschool psychopathology. *J. Child Psychol. Psychiatry* 48 1005–1013. 10.1111/j.1469-7610.2007.01770.x 17915001 PMC2853244

[B59] Sterba S. K. Prinstein M. J. Cox M. J. (2007). Trajectories of internalizing problems across childhood: heterogeneity, external validity, and gender differences. *Dev. Psychopathol.* 19 345–366. 10.1017/S0954579407070174 17459174

[B60] Thomas A. Chess S. (1977). *Temperament and Development.* Oxford: Brunner/Mazel.

[B61] Thomas J. Jones G. Scarinci I. Brantley P. (2003). A descriptive and comparative study of the prevalence of depressive and anxiety disorders in low-income adults with type 2 diabetes and other chronic illnesses. *Diabetes Care* 26 2311–2317. 10.2337/diacare.26.8.2311 12882854

[B62] Tucker P. van Zandvoort M. M. Burke S. M. Irwin J. D. (2011). Physical activity at daycare: childcare providers’ perspectives for improvements. *J. Early Child. Res.* 9 207–219. 10.1177/1476718X10389144

[B63] U.S. Department of Health and Human Services [HHS] (2017). *President’s Council on Fitness, Sports & Nutrition: Facts & Statistics.* Available at: https://health.gov/paguidelines/second-edition/pdf/Physical_Activity_Guidelines_2nd_edition.pdf

[B64] Vierhaus M. Rueth J. E. Lohaus A. (2018). The observability of problem behavior and its relation to discrepancies between adolescents’ self-report and parents’ proxy report on problem behavior. *Psychol. Assess.* 30 669–677. 10.1037/pas0000515 28782976

[B65] Wichstrøm L. Berg-Nielsen T. S. Angold A. Egger H. L. Solheim E. Sveen T. H. (2012). Prevalence of psychiatric disorders in preschoolers. *J. Child Psychol. Psychiatry* 53 695–705. 10.1111/j.1469-7610.2011.02514.x 22211517

[B66] Wonderlich S. J. (2007). *Predictors of Bullying Behaviors: A Follow-Up Study on Early Childhood Play Behaviors of Preschoolers.* Master’s thesis, Southern Illinois University, Carbondale, IL.

[B67] Woods S. B. Farineau H. M. McWey L. M. (2012). Physical health, mental health, and behaviour problems among early adolescents in foster care. *Child Care Health Dev.* 39 220–227. 10.1111/j.1365-2214.2011.01357.x 22329484

[B68] World Health Organization [WHO] (2004). *Healthy People 2010: Final Review.* Available at: https://www.cdc.gov/nchs/data/hpdata2010/hp2010_final_review.pdf

[B69] World Health Organization [WHO] (2013). *Mental Health of Older Adults, Addressing a Growing Concern.* Available at: http://www.who.int/mental_health/world-mental-health-day/WHO_paper_wmhd_2013.pdf

[B70] Zach S. Zeev A. Dunsky A. Goldbourt U. Shimony T. Goldsmith R. (2013). Adolescents’ physical activity habits–results from a national health survey. *Child Care Health Dev.* 39 103–108. 10.1111/j.1365-2214.2012.01392.x 22676356

